# Lack of Association between Cu T-380A Intrauterine Device and Secondary Infertility in Iran

**DOI:** 10.22074/ijfs.2016.5085

**Published:** 2016-11-01

**Authors:** Mahnaz Abdinasab, Razieh Dehghani Firouzabadi, Tahmineh Farajkhoda, Ali Mohammad Abdoli

**Affiliations:** 1 Yazd Research and Clinical Center for Infertility, Shahid Sadoughi University of Medical Sciences, Yazd, Iran; 2 Research Center for Nursing and Midwifery Care, Shahid Sadoughi University of Medical Sciences, Yazd, Iran

**Keywords:** Copper Intrauterine Devices, Infertility, Complication, Cohort Study, Iran

## Abstract

**Background:**

The appropriate choice of a contraceptive method has been a major issue in reproductive health research. Cu T intrauterine device (Cu T IUD) has been introduced as one of the most effective contraceptive methods in the world, however, the relationship between prior use of Cu T IUD and secondary infertility has not been evaluated in Iran. To examine the association of Cu T-380A IUD and secondary infertility in Iran.

**Materials and Methods:**

A retrospective cohort study was conducted from December 2010 to September 2011 in the Research and Clinical Center for Infertility, Shahid Sadoughi University of Medical Sciences, Yazd, Iran. A total of 750 married women (15-49 years old) with at least one parity, whom were referred to four educational healthcare centers of Shahid Sadoughi University of Medical Sciences, were selected as participants. They were divided into two groups (case and control) based on previous history of using Cu T-380A IUD. Data were gathered using a standard reliable questionnaire along with a face-to-face interview and were analyzed with descriptive and analytical (χ²) tests.

**Results:**

Mean period of Cu T-380A IUD usage in the case group was 57.46 ± 47.74 months and mean time length from Cu T-380A IUD removal to pregnancy was 14.87 ± 5.18 months in this group. We observed no relationship between the use of Cu T-380A IUD and frequency of secondary infertility (3.5% in the case group versus 2.7% in the control group, P=0.52).

**Conclusion:**

Given the relatively large sample size studied here, it is unlikely that Cu T-380A IUD results in secondary infertility and may be used by Iranian women as a safe contraceptive method.

## Introduction

The Copper T-380A intrauterine device (Cu T- 380A IUD) is one of the most effective, long-term and reversible contraceptive methods worldwide (1, 2). The American College of Obstetricians and Gynecologists (ACOG) has established Cu T- 380A as a safe contraception (2). Other notable advantages of this method are low-cost, convenience and its acceptability among women (3, 4).

According to the International Planned Parenthood Federation (IPPF), more than 95% of women who use CU IUDs are satisfied with them (5-7). It is, however, important to note that the Cu T- 380A does not protect against sexually transmitted diseases (STDs) and pelvic inflammatory disease (PID) (6-8). Several studies have reported an association between the use of CU IUDs and secondary infertility following STDs or PID in parous women (3, 8-12). For instance, a double risk of tubal infertility has been associated with prior use of CU IUD in two independent studies (8, 9). On the contrary, Hubacher et al. (11) showed in a well-designed case-control study that prior use of CU IUD was not associated with subsequent infertility. Several studies have shown the rare but serious side effects of CU IUDs such as STDs or PID when used in a monogamous relationship (6, 7, 12). There are also misconceptions about secondary infertility after removing CU IUD in the mind of women who use this method (13-15). According to Mansour et al. (16), fear of subsequent infertility following the use of CU IUD is considered as one of the main concerns in use of IUD among women and thought to be one of the most important factors for cessation of IUD use in developing countries (15) where it is commonly used (17). Islamic Republic of Iran has seen significant achievements in family planning in recent years (18, 19) with the use of contraception increasing from 49.9% in 1989 to 73.8% in 2009 (20, 21). This mainly has been driven by free healthcare services for family planning methods such as Cu T-380A IUD throughout the Public Health Center Network (20). The national research of Integrated Monitoring Evaluation System in Iran (IMES) reported IUD users to comprise 11.9% of contraceptive users in Iran (22).

The prevalence of infertility is high in Iran at nearly 20% (23). While this has been reported about 10-15% worldwide (23-29). The notable percentage of Cu T-380A IUD usage by Iranian women (21), specific socio-cultural background of Iranian family regarding female infertility and cultural issues of child adoption among Iranian families (30) and lack of enough study in this regard, led us to examine the association of Cu T-380A IUD and secondary infertility in the Yazd province.

## Materials and Methods

**Fig.1:**
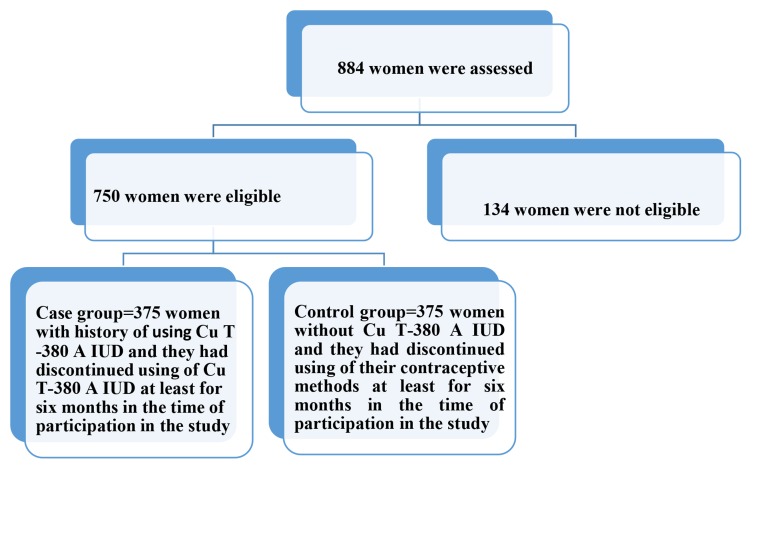
Sampling process for selecting the case and control groups. IUD; Intrauterine device.

We gathered current registry information under A retrospective cohort study was carried out from December 2010 to September 2011 in the Research and Clinical Center for Infertility, Shahid Sadoughi University of Medical Sciences, Yazd, Iran. The Ethics Committee of Shahid Sadoughi University of Medical Sciences of Yazd approved this study (code number#823). The study was conducted in four educational health care centers (Akbari, Rahmat Abad, Maskan and Azad Shahr) of Shahid Sadoughi University of Medical Sciences of Yazd. Data regarding secondary infertility and use of Cu T-380 A IUD were gathered by a face-to-face interview along with a structured questionnaire. The sampling process has been illustrated in the flowchart below (Fig .1).

After obtaining written informed consent, 750 married women in reproductive age (15-49 years old) attending one of the aforementioned healthcare centers with a history of using contraceptive methods including Cu T-380A IUD, combined oral contraceptive pill (OCP), withdrawal method and male condom, were selected as participants through conducting convenience sampling method. One hundred thirty four women were not eligible because they had not inclusion criteria or had not consent or time to participate in the study. Women with a history of using only progestin methods such as minipills, depotmedroxyprogesteroneacetate (DMPA) and Minera IUD were not eligible for participation in the study. All the inclusion criteria were a history of using Cu T-380A IUD for at least six months (only in the case group) and discontinued use of Cu T-380 A IUD for at least six months at the time of participation in the study, having regular intercourse, history of at least one pregnancy, no history of primary infertility or infertility treatments, no systematic diseases, and also no use of additional contraceptive methods at the time of sampling. Eligible women who withdrew from the study after participation were taken into account as the exclusion criterion. Participants had discontinued using their aforementioned contraceptive methods for at least six months at the time of participating in this study. The following formula shows sample size calculation.

In this study P_1_=Prevalance in study population, P_2_=Variance of study population, α=The probability of making a type I error, N=Total sample size, n_1_=Sample size in group 1 and n_2_=Sample size in group 2. This formula is used for calculation of sample size for qualitative variable such as occurnece of pregnancy in two groups in this study.

P_1 (Prevalance)_=2%

P_2 (Variance)_=36%

α (The probability of making a type I error)=5%

N=Total sample size

n_1 (Sample size in group 1)_=375

n_2 (Sample size in group 2)_=375

$$N=Z_{1-\frac{\alpha }{2}\mathrm{P1}(1-\mathrm{P1})+\mathrm{P2}(1-\mathrm{P2})}^{2}$$

Women were divided into two groups (case group=375 women and control group=375 women) based on prior history of using Cu T-380A IUD. Participants were matched for age (+/2 years). The control group had a history of using other contraceptive methods such as OCP, withdrawal method and male condom. Data were gathered by a standard structured questionnaire based on scientific literature review. Experts in the related discipline approved content validity of the questionnaire (CVI=0.91). Reliability (internal consistency) of the questionnaire was calculated using Cronbach’s alpha statistical test (α=0.89). The questionnaire consisted of various questions regarding personal characteristics, reproductive history and use of Cu T-380A IUD, and were completed in a face-to-face interview. Additional data were obtained from previous records in healthcare centers. Age, parity, length of using previous contraceptive methods before participation in the study, problems during use of contraceptive methods, complications needing treatment, response to treatment, hospitalization for PID, control visits for PID fallow up, date of discontinuing contraceptive methods and reasons of withdrawing were obtained from records and compared with participant answers. Also, occurrence of pregnancy was asked from the participants as and then it checked in their medical record as a variable to measure study outcome. Since recall bias is common and an inevitable factor in retrospective studies, we used both methods of data gathering and compared accuracy of obtained data from participants by the quesionnaire. History of PID was also gathered from records and questionnaires. Two questions were asked from participants such as “do you have a history of uterine infection so serious you had to behospitalized?” and “do you have a history of uterine infection with fever, infected vaginal discharge and pain in the pelvic area so serious that you had to visit a gynecologist and receive injectable or oral antibiotics?”

### Statistical analysis

Data were analyzed by SPSS (version 16) using descriptive (mean and SD) and analytic (χ²) statistical tests. Significance level was considered at P<0.05.

## Results

All 750 women completed the questionnaires in full. Mean age difference between the case group (34.79 ± 7.41 years) and the control group (33.93 ± 7.77 years) was not statistically significantly different (P=0.123). The results showed that there were significant statistical differences in mean number of gravidity (P=0.001) and abortion (P=0.038) in the case and control groups. Mean length of Cu T-380A IUD in the case group was 57.46 ± 47.74 months and mean length from Cu T-380A IUD removal to pregnancy was 14.87 ± 5.18 months (Table 1).

**Table 1:** Comparison of personal characteristics in the case and control groups


Groups	Case(mean ± SD)	Control(mean ± SD)	P value	

Age (Y)t test	34.79 ± 7.41	33.93 ± 7.77	P=0.123	
Number of gravidityt test	3.12 ± 1.07	3.60 ± 1.58	P=0.001^*^	
Number of abortiont test	0.33 ± 0.75	0.23 ± 0.59	P= 0.038^*^	
Mean length of Cu T-380A IUD using (month)	57.46 ± 47.74		
Mean length from Cu T-380A IUD removal to pregnancy (month)	14.87 ± 5.18		

**Educational status**	**Casen (%)**	**Controln (%)**	**Total**	**P value**

Under diploma	200(53%)	244(65%)	444(59%)	P=0.002^*^
Diploma and greater	175(47%)	131(35%)	306(41%)	
Total	375(100%)	375(100%)	750(100%)	

**Occupational status**	

Housewife	327(87%)	338(90%)	665(89%)	P>0.05
Employee	48(13%)	37(10%)	85(11%)	
Total	375(100%)	375(100%)	750(100%)	


^*^; Statistically significant.

A significant statistical difference was observed in educational level between the case and control groups (P=0.002) but not in occupational status (P>0.05, Table 1).

Prior history of PID was observed in 30 women (8.1%) in the case group and in 26 women (7.1%) in the control group, with the difference not statistically significant [P=0.61, odda ratio (OR)=0.867, confidence interval (CI)=(0.50-1.49), Table 2].

**Table 2:** Comparison of prior history of pelvic inflammatory disease (PID) based on both self-reported data by women and their clinical records in the case and control groups


Groups	Case(n=375)	Control(n=375)	Total

With Prior history of PID	30	26	56
	8%	6.9%	7.6%
Without Prior history of PID	345	349	694
	92%	93%	92.4 %
Total	375	375	750
	100%	100%	100%


The main aim of this study was to examine the relationship between the use of Cu T-380A IUD and secondary infertility. The results showed that there was no signifiacnt association between history of Cu T-380 A IUD use and secondary infertility [3.5% in the case group versus 2.7% in the control group, P=0.52, OR=1.31, CI=(0.57-3.03), Table 3].

**Table 3:** Comparison of secondary infertility based on occurrence of pregnancy at least six months after discontinuing the contraceptive method in the case and control groups


Groups	Case(n=375)	Control(n =375)

No occurrence of pregnancy	13	10
	3.5%	2.7%
Occurrence of pregnancy	362	365
	96.5%	97.3%
Total	375	375
	100%	100%


There was also no significant difference between the case and control groups (P=0.5) in perceiving an association between use of Cu T-380A IUD and secondary infertility, with women equally divided between agreeing, disagreeing and not knowing (Table 4).

**Table 4:** Comparison of perception of women regarding the association between prior use of Cu T-380A intra utrine device (IUD) and secondary infertility


Groups	Case	Control	Total

I agree that prior use of Cu T-380A IUD can cause secondary infertility	133	128	261
	35.5%	34.1%	
I disagree agree that prior use of Cu T-380A IUD can cause secondary infertility	118	114	232
	31.4%	30.4%	
I don’t have any idea concerning association between prior use of Cu T-380A IUD and secondary infertility	124	133	257
	33.1%	35.5%	
Total	375	375	750


## Discussion

Conducting reproductive health research (RHR), should be done in safe practice manner and women should fulfill the benefits of scientific progress in the community including contraceptive research (31-33). The aim of this study was to assess the relationship between use of Cu T-380A IUD and secondary infertility. We observed no such association. Philippov et al. (29) showed that the prevalence of primary and secondary infertility respectively after Cu T IUD removal was 2.6 and 10.8%. Mansour et al. (16) assessed a thorough literature review for prospective studies reporting pregnancy rates in women after discontinuing contraceptive methods. One-year pregnancy rate following removal of Cu T-380A IUD was reported high, ranging from 71 to 91%, similar to other barrier methods or use of no contraceptive method. In another study, the observed fertility rate following cessation of use of Cu IUDs implied that fertility is normal after discontinuation of this method (34). These findings are consistent with those of this study.

We found the frequency of prior history of PID to be similar in the case and control groups. Several studies have assessed association between Cu IUD use and PID or salpingitis (7-9). Daling et al. (8) and Cramer et al. (9) reported a doubling in the risk of tubal infertility associated with prior use of IUD. They reported that there was not any risk of secondary infertility in women with prior use of CU IUDs even the copper device had been removed for the reason of CU IUDs complications. In this regard, Cramer et al. (9) reported that the number of partners is as an important factor, since women with only one sexual partner had no significant increase in the risk of tubal infertility, regardless of the type of contraception used. Lack of bacteriologic information in both case-control studies was an important limitation. Hubacher et al. (11), however, undertook a well-designed case-control study to determine the risk of tubal sterility associated with CU IUD use. Their results showed that use of CU IUD was not associated with subsequent infertility. They reported that history of prior infection with Chlamydia trachomatis (confirmed by antibodies against Chlamydia) was significantly associated with tubal infertility. Incontrast, use of a CU IUD showed no association with tubal infertility. These two findings showed that STDs (chlamydial infection and gonorrhea, particularly), and not plastic or copper, are the common causes of tubal infertility. Bromham (35) reported that when one controls for the confounding effect of prior STD exposure, the increase in risk associated with CU IUD disappears. Hamerlynck and Knuist (13) believe that resistance against the use of CU IUDs due to the perceived increased risk of PID and subsequent infertility is related to lack of awareness with respect to recent developments and also unpleasant experiences during the 1980s and earlier when Dalkon Shield IUDs were used. According to Huggins and Cullins (12), the risk of infertility associated with prior use of CU IUD is low. PID and its consequences are primarily a concern in the immediate post-insertion time frame or to women who have a greater risk of acquiring STDs (those with multiple partners or those with prior PID). Also, Swende et al. (17) have shown that contamination at the time of IUD insertion is responsible for PID. The excess risk of PID among IUD users, with the exception of the first few months after insertion, is related to STDs and not the IUD. Women without risk factors for STDs have little increased risk of PID or infertility associated with IUD use (12). Furthermore, Kohn et al. (2) showed that 31% of all healthcare providers recommend an IUD for clients with history of recent STD, 37% of them suggest IUD insertion for women with previous history of PID and 38% for clients not in a monogamous sexual relationship. Although 77% of healthcare providers stated that IUDs were safe for adolescents, it would be unlikely for 18% of them to recommend an IUD to a client under 20 years of age. Moreover, 86% of respondents were aware that IUDs can be used in nulliparous women, however, 25% of them would not recommend an IUD to such clients. There are no contraindications for IUD use based solely on age or parity (2, 36).

Assessing the view of women on prior use of Cu T-380A IUD and its association with secondary infertility was one of aims of this study. Although association between prior use of Cu T-380A IUD and secondary infertility was not observed between the case and control groups but 261 women from 750 women who participated in the study believed that prior use of Cu T-380A IUD can cause secondary infertility. It seems that women need to be better educated on this issue. Stoddard et al. (4) suggested that IUD should be offered as the first-line contraceptive method for most women and the misconceptions regarding IUD should be explained to women. In another study, 61% of healthcare providers believed that counseling clients about IUD would take more time than other methods but it is necessary (2). Given that recent studies have shown that Cu IUDs maybe used by eligible nulliparous women and adolescent girls (15, 37), they have been introduced as a safe contraceptive method for such individuals which have no risks factor for STDs and PID (2, 36).

The high prevalence of infertility threatens familial life, health and social status of women in our country. Primary, secondary and lifetime infertilities have been reported to be at 21.1, 7.8 and 6.4%, respectively in population-based study of Iran (38). Since prevalance of infertility is high in Iran (23, 38), the most important reason of primary and secondary infertilities is menstrual dysfunction (not use of Cu T-380A IUD). The socio-demographic factors can influence the prevalence of infertility. Providing information about significant factors on infertility may help women to increase their fertility chances (38). In developing countries like Iran, having children is greatly valued for social, cultural and economic reasons (39). From an Islamic perspective, attitude toward motherhood is extremely honored with the famous tradition stating that "heaven lies at the feet of mothers" (40). Since infertility may prevent couples to attain the desired life and cause some social and psychological problems, Iranian women with infertility may undergo several individual and social problems that may influence their quality of lives.

Given that we identified no association between prior use of Cu T-380 A IUD and occurrence of secondary infertility, introducing this method as a long-acting contraceptive for eligible women can help women and their husbands to decrease their stress level of secondary infertility and have a pleasant experience with their selected contraceptive method.

## Conclusion

We observed no association between prior use of Cu T-380A IUD and occurrence of secondary infertility. Women may use currently available Cu T-380A IUD without any fear of secondary infertility. However, it should be kept in mind that this is a good contraceptive choice for eligible women only. Overall, it is recommended that the risks and advantages of using the method should be considered on an individual basis. One of the best ways for selecting eligible women for IUD use is for individuals to undergo appropriate counseling. Barriers such as pervasive myths, misconceptions, misinformation and incorrect beliefs among healthcare providers, clients and communities should be explained briefly.
